# Identification and characterization of a subpopulation of CD133^+^ cancer stem‐like cells derived from SK-UT-1 cells

**DOI:** 10.1186/s12935-021-01817-y

**Published:** 2021-03-08

**Authors:** Jiuping Gao, Ting Yang, Xu Wang, Yi Zhang, Jing Wang, Beilei Zhang, Dihong Tang, Yanqiong Liu, Ting Gao, Qiuhui Lin, Jun Tang, Jingting Cai

**Affiliations:** 1 Department of Gynecological Oncology, The Affiliated Tumor Hospital of Xiangya Medical School of Central South University, Changsha, China; 2 Department of Gynecology and Obstetrics, Xiangya Hospital Central South University, Changsha, China; 3 Department of Gynecology and obstetrics, The second people’s Hospital of Hunan Province, Changsha, China; 4 Department of Gynecology and obstetrics, Central Hospital of Yiyang City, Yiyang, Hunan Province China; 5 Department of Gynecology and Obstetrics, The First People’s Hospital of Shaoguang, Shaoguan, Guangdong Province China

**Keywords:** Uterine leiomyosarcoma, Cancer stem cells, Tumorspheres, Drug resistance, CD133

## Abstract

**Background:**

Uterine leiomyosarcoma (ULMS) is a malignant tumor found in the smooth muscle lining the walls of the uterus. Cancer stem cells (CSCs) are responsible for metastasis, drug resistance, and relapse of cancer, resulting in treatment failure. However, little is known about CSCs and their associated-markers in ULMS. We aimed to characterize and identify a subpopulation of CD133^+^ cancer stem-like cells derived from SK-UT-1 cell line.

**Methods:**

SK-UT-1 cells were sphere-forming cultured in vitro. We also sorted the CD133^+^ cells derived from SK-UT-1 cell line by immunomagnetic beads. CD133^+^ subpopulation and apoptotic cells were detected by flow cytometry. Self-renewal and anchorage-independent growth capabilities were examined using sphere and colony formation assays. The tumorigenicity of the fourth-passage spheres and parental SK-UT-1 cells was used by mouse xenograft model in vivo. Cell proliferation ability and sensitivity to doxorubicin (DXR) were assessed by CCK-8 assay. Cell migration and invasion were tested by wound healing assay or Transwell migration and invasion assays. Expressions of CSC-related marker were analyzed by Western blotting.

**Results:**

The fourth-passage spheres were defined as a CD133^+^ cell population, which was accompanied by increase of sphere and colony forming rate, migration and invasion abilities, as well as drug-resistant properties in vitro. Moreover, the fourth-passage spheres showed a stronger tumorigenic potential in vivo. CD133^+^ cell population sorted from SK-UT-1 line showed an increased ability in sphere and colony formation, proliferation, migration, invasion, resistance to apoptosis after treatment with doxorubicin (DXR) compared with CD133^−^ cell population. The expression levels of CSCs-related markers (e.g., CD44, ALDH1,BMI1, and Nanog), were significantly elevated in CD133^+^ cells compared with those in CD133^−^ cells.

**Conclusions:**

Collectively, our findings indicated that CD133 may be a significant marker for cancer stem-like cells, and it may be a potential therapeutic target for human ULMS.

## Background

Uterine leiomyosarcoma (ULMS) is an aggressive malignancy characterized by its early metastasis, high rates of recurrence, and poor prognosis [1]. The response rate to chemotherapeutic drugs, such as paclitaxel and cisplatin, is as low as 18 %. To date, the recurrence rate of ULMS remains as high as 70 % [2, 3]. Therefore, it is highly essential to explore and clarify the mechanisms underlying the growth, metastasis, recurrence, and drug resistance of ULMS.

Cancer stem cells (CSCs) are cancer cells that possess characteristics associated with normal stem cells, and they may generate tumors through the stem cell processes of self-renewal and differentiation into multiple cell types [4]. CSCs are responsible for metastasis, drug resistance, and relapse of cancer, resulting in treatment failure [5]. Meanwhile, these cells highly express surface markers similar to those of normal stem cells, including CD44, CD24, and CD133 [6]. However, little is known about CSCs and their associated-markers in ULMS.

CD133, a transmembrane glycoprotein also known as prominin-1, is normally expressed on undifferentiated cells including endothelial progenitor cells [7], hematopoietic stem cells [8], fetal brainstem cells [9], and prostate epithelial cells [10]. Several studies have used CD133 as a marker to identify CSCs [11–17]. In the present study, we, for the first time, characterized and identified a subpopulation of CD133^+^ cancer stem-like cells derived from SK-UT-1 (a human ULMS cell line), and demonstrated that CD133 may be as a significant marker for cancer stem-like cells, highlighting its potential role in the treatment of human ULMS.

## Materials and methods

### Culture of SK-UT-1 cells and spheres

SK-UT-1 cell line was obtained from the American Type Culture Collection (Manassas, VA, USA), and maintained in Dulbecco’s modified Eagle’s medium (DMEM) (Hyclone Laboratories Inc., Marlborough, MA, USA) containing 10% fetal bovine serum (FBS; Gibco Laboratories, Gaithersburg, MD, USA), 1 % penicillin and streptomycin at 37 °C in presence of 5 % CO_2_. For tumorsphere culture, suspended single cells were cultured at a density of 2 × 10^5^ cells/well in 6-well ultra-low cell-attachment plates (Corning Inc., Corning, NY, USA) and grown in cancer stem cell medium (CSC-M, namely DMEM/F12 medium containing 20 ng/ml epidermal growth factor (PeproTech, Rocky Hill, NJ, USA), 20 ng/ml basic fibroblast growth factor (PeproTech, Rocky Hill, NJ, USA), 2% B27 (Invitrogen, Carlsbad, CA, USA), 4 µg/mL bovine serum albumin (Dingguo Changsheng Biotechnology Co., Ltd., Beijing, China), and 4 µg/mL insulin (Wanbang Biopharmaceuticals Co., Ltd., Xuzhou, China) at 37 °C in presence of 5% CO_2_. Once the tumorspheres (diameter ≥ 50 µm) formed, cells were passaged approximately every 6 days by centrifugation, removal of supernatant, resuspension, and plating as mentioned above.

### Determination of sphere formation rate

The primary spheres and the fourth-passage sphere-derived cells or CD133^+^ cells or CD133^−^ cells were seeded into 24-well ultra-low cell-attachment plates at a density of 1000 cells/well. After that, the cells were cultured for 6 days, and the number of tumorspheres per well was counted. Sphere formation rate was calculated as follows: sphere formation rate (%) = the number of formed spheres per well/total number of cells inoculated (1000 cells) × 100 %.

### Limiting dilution analysis

The primary passage spheres and the passage 1–4 spheres were dissociated as describe above, and 100 cells were plated in 150 µl of growth medium in a 96-well culture plate to obtain a single cell per well. 20 µL of growth medium was added to each well every 2 days. The number of tumorspheres in each 96-well culture plate was counted after 6 days of cultivation.

### Recording time course required for sphere formation

The dissociated single sphere-forming cells were diluted to a density of 500 cells/mL. Then, 2 µl/well diluted cell suspension was seeded into ultra-low cell-attachment 96-well plate (Corning Inc., Corning, NY, USA), and 150 µl of CSC-M was added. The wells that contained one single cell were marked and monitored every day.

### Flow cytometry assay

The primary spheres or spheres at passages 1–4 were collected and resuspended to obtain single cell suspensions, respectively. After washing with ice-cold phosphate-buffered saline (PBS) and centrifugation at 1000 rpm for 5 min, cells were collected and resuspended at 1 × 10^6^ cells/mL in a culture medium. Cells were then stained with fluorescence-conjugated antibody against CD133 (1:100; Bioss, Beijing, China) or isotype IgG (Bioss, Beijing, China) as a control. Cytometry analyses were performed using a FACS Vantage flow cytometer (Becton, Dickinson and Company, Franklin Lakes, NJ, USA) and Cytomation Summit software. The experiment was repeated for 3 times, and data of 3 independent experiments were expressed as mean ± standard deviation (SD).

For cell apoptosis, the CD133^−^ and CD133^+^ population derived from SK-UT-1cell line were collected and resuspended to obtain single cell suspensions, respectively. Cells were then seeded into 6-well plates at a density of 1 × 10^6^ cells/well and treated with doxorubicin (DXR) at a concentration of 10 µM on the following day. After 24 h of incubation, cells were collected and centrifuged at 1000 rpm for 8 min followed by 3 times washing with ice-cold PBS. Then, 1 × 10^5^ cells were resuspended in 50 µL of staining buffer and incubated with 5 µL of propidium iodide (PI) and 5 µL of AnnexinV-FITC in dark for 15 min, then analyzed using a FACS Vantage flow cytometer (Becton, Dickinson and Company, Franklin Lakes, NJ, USA). The experiment was repeated for 3 times, and data of 3 independent experiments were expressed as mean ± SD.

### Anchorage‐independent growth assays

Cells were suspended in CSC-M containing 0.3% soft agar to carry out colony formation assay. After 2 weeks, formed colonies (≥ 20 cells) were counted under a microscope and representative images were photographed.

### Transwell migration and invasion assays

Cell migration assay was performed in a 24-well plate using Transwell inserts (Corning Inc., Corning, NY, USA) with 8.0-µm pore size. The primary spheres and spheres at 4 passage were collected and resuspended in DMEM and CSC-M, respectively. Then, 200 µL of growth factor-free CSC-M containing 5 × 10^4^ cells was loaded onto the upper chamber of the transwell insert, and 600 µL of CSC-M was loaded onto the lower chamber. Cells were allowed to migrate for 24 h at 37 °C. The inserts were washed with PBS, and non-migratory cells that remained in the upper compartment were removed with a cotton swab. The migratory cells in the lower chamber were fixed with methanol and stained with crystal violet (Hengxing Chemical Reagent Co., Ltd., Tianjin, China), and counted using an optical microscope (magnification, x400; Olympus, Tokyo, Japan). Cell invasion assay was performed using matrigel-precoated (Becton, Dickinson and Company, Franklin Lakes, NJ, USA) transwell chambers (8-µm pore size; Corning Inc., Corning, NY, USA) in a 24-well plate.

### Cell counting kit-8 (CCK-8) assay

To demonstrate the viability of CD133^−^ or CD133^+^ population drived from sK-UT-1 and half maximal inhibitory concentration (IC_50_) value for DXR, cells were treated with different concentrations of DXR (0, 0.5, 1.0, 2.0, 4.0, 8.0, 16.0, 32.0, 64.0, and 128.0 µM) for 48 h. 10 µL of CCK8 reagent was added to 96-well plates, and incubated with CCK8 reagent for an additional 2 h. The absorbance was measured by a microplate reader at wavelength of 450 nm.

The proliferation ability of CD133^−^ or CD133^+^ cells was assessed by CCK-8 assay. For this purpose, 2 × 10^3^ cells in 100 µl cell suspension were seeded into the 96-well plates and cultured for 24 h. After that, cells were treated with 10 µL of CCK-8 solution (Dojindo, Tokyo, Japan) and absorbance at 450 nm was detected at 0, 24, 48, 72, and 96 h, respectively.

### Western blotting

Western blotting was carried out according to the manufacture’s protocol. The following primary antibodies including anti-CD44, anti-ALDH1, anti-BMI1, anti-Nanog, anti-ABCG2, anti-OCT4 (Abcam, Cambridge, UK), and anti-β-actin (Santa Cruz Biotechnology, Inc. Dallas, TX, USA) were used. Horseradish peroxidase (HRP)-conjugated antibodies (Beyotime Institute of Biotechnology,Shanghai, China) were used as secondary antibodies. Blots were detected using an enhanced chemiluminescence (ECL) reagent (GE Life Sciences, Piscataway, NJ, USA).

### Mouse xenograft model

Six-week-old BALB/c-nu female mice weighing 18–22 g were purchased from Beijing Vital River Laboratory Animal Technology Co., Ltd. (Beijing, China) and maintained under specific-pathogen-free conditions at Xiangya Hospital, Central South University (Changsha, China). All the animal experiments were approved by the Animal Ethics Committee of Xiangya Hospital. Mice were randomly divided into 4 groups (n = 5/group) and were injected subcutaneously with resuspended 10^4^, 10^5^, and 10^6^ SK-UT-1 cells derived from spheres into the right flank of each mouse. Next, 10^5^, 10^6^, and 10^7^ parental SK-UT-1 cells were injected into the left symmetric flank of the same mouse. After the initial appearance, tumors were measured every day using a caliper. Tumor volume was calculated as V = ½ (length × width^2^).

### Cell sorting using immunomagnetic beads

SK-UT-1 cells were resuspended with 300 µl PBS at a density of 1 × 10^7^ cells/ml and sorted using the CD133 MicroBead kit (Miltenyi Biotec GmbH, Bergisch Gladbach, Germany) according to the manufacturer’s instructions. Following this, cells were divided into three groups (SK-UT-1, CD133–, and CD133 + groups), and homotypic IgG antibody served as control. Flow cytometry was used to detect the percentage of CD133^+^ cells in each group.

### Wound healing assay

For wound healing assay, cells were seeded into 6-well plates until reaching full confluence in a monolayer. A micro-pipette tip was utilized to scratch a single wound in each well. The plate was incubated at 37 °C in presence of 5% CO_2_. Images were taken at 0 and 24 h after scratching and were subsequently analyzed. The average cell migration rate was expressed as relative width of wound/time. All experiments were carried out in duplicate.

### Statistical analysis

All experiments were repeated for at least 3 times. Data were expressed as mean ± SD. Two-tailed Student’s *t*-test was used for comparing differences between two groups, and two-way analysis of variance was employed for comparing differences among multiple groups. GraphPad Prism 8.0 software (GraphPad Software, Inc., La Jolla, CA, USA) was used to statistically analyze data. *P* < 0.05 was considered statistically significant.

## Results

### Sphere‐forming culture could enrich CD133^+^ cells derived from SK-UT-1 cells

**Fig. 1:**
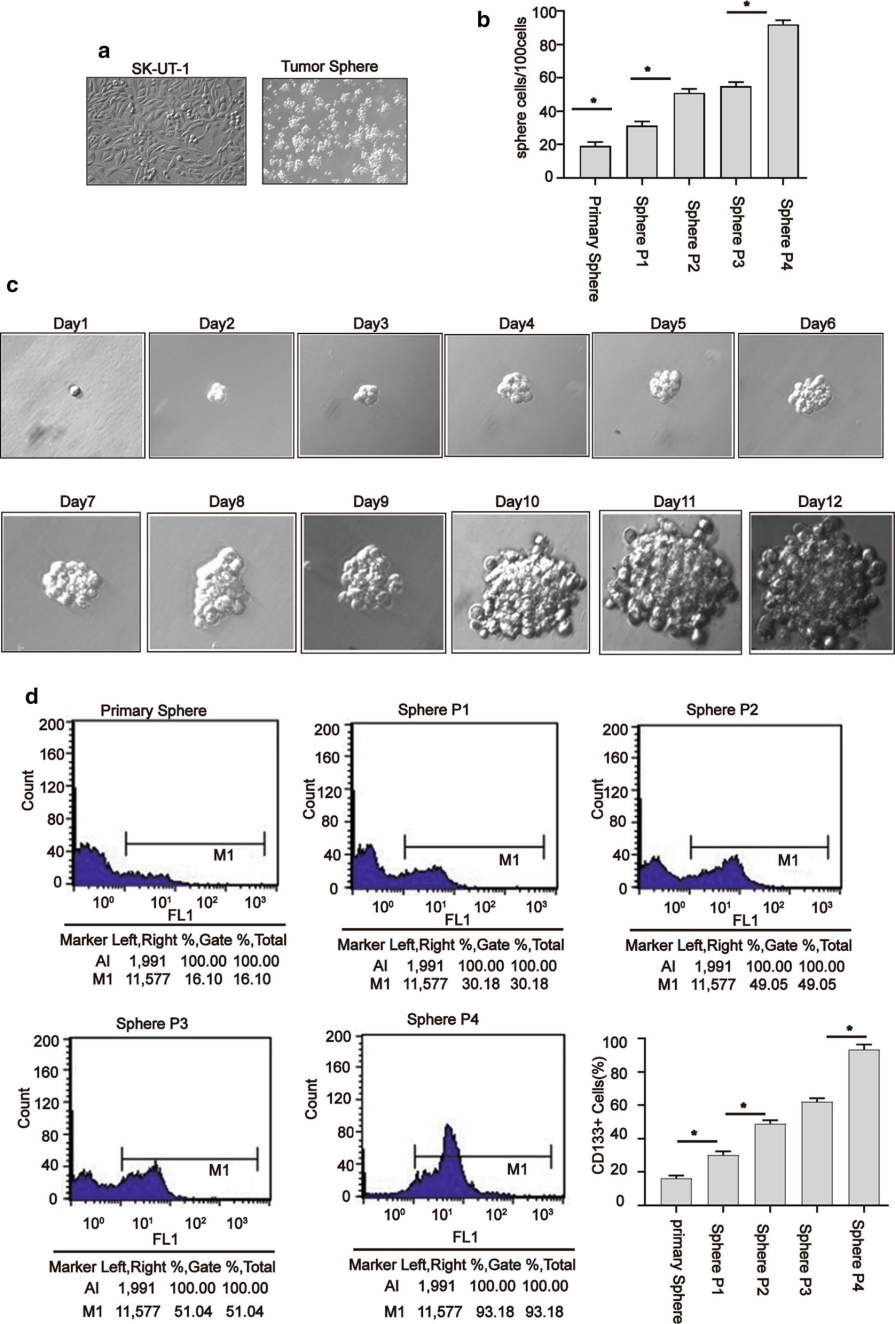
Sphere culture caused enrichment of CD133 ^+^ population. The morphology of parental SK-UT-1 cells and spheres (magnification, ×200) (**a**), sphere formation rate of primary sphere and spheres at passages 1–4 on the 6 day (**b**), the process of formation of spheres derived from the SK-UT-1 cells (magnification, ×400) (**c**), SK-UT-1 derived-spheres enriched CD133 cells (**d**). The primary spheres and spheres at passages 1–4 were stained with FITC-conjugated antibody against CD133 and analyzed using a flow cytometer. Data were presented as mean ± SD (n = 3, *P < 0.05)

Sphere-forming culture is often used to enrich CSCs or cancer stem-like cells [18, 19]. To explore whether there is a small fraction of cells with stem cell-like characteristics in SK-UT-1 cells, we performed sphere-forming culture. Figure 1a showed the morphology of SK-UT-1 cells and spheres under a microscope (magnification, x200).

We next used a limiting dilution assay to examine the ability of single cells from the primary spheres and spheres at 1–4 to produce new spheres. Our findings revealed that the sphere-forming rate of fourth-passage spheres on 6 days was higher than that of the other passages of spheres (Fig. 1b).

To corroborate the findings that a sphere could be generated from a single cell, one fourth-passage sphere cell was plated to a 96-well plate, and wells with one cell were daily visualized. Figure 1c illustrates the process of formation of spheres derived from the SK-UT-1 cells.

In order to indicate whether sphere-forming tumor cells possess properties of cancer stem-like cells, we detected CD133 expression in different passage spheres. It was revealed that the percentage of CD133^+^ cells in the fourth-passage spheres was more than 90% (Fig. 1d), and we used it for subsequent assay. Collectively, our findings indicated the fourth-passage spheres derived from SK-UT-1 cells could enrich CD133^+^ cell population.

### Fourth-passage spheres enhanced characteristics of stem cell-like cells in SK-UT-1 cells

**Fig. 2:**
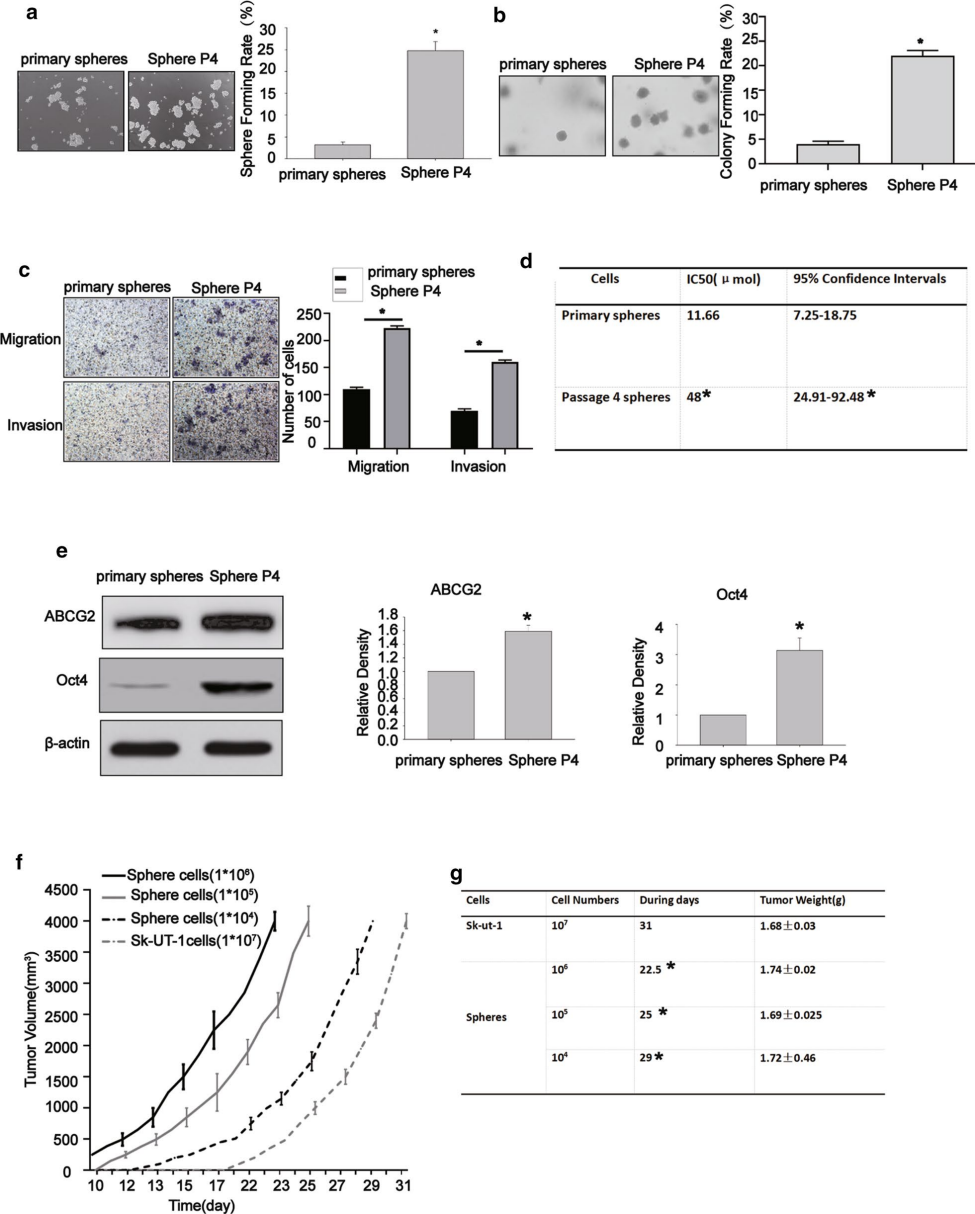
The fourth-passage spheres enhanced carcinogenicity in vitro and in vivo. The fourth-passage spheres showed higher sphere formation rate than primary-passage spheres (**a**), the fourth-passage spheres showed higher colony formation rate than primary-passage spheres (**b**), the fourth-passage spheres exhibited higher migration and invasion rates (**c**), the IC_50_ of primary-passage spheres and the fourth-passage spheres (**d**), the relative density of primary-passage spheres and the fourth-passage spheresof ABCG2 and Oct4 (**e**), Data were presented as mean ± SD (n = 3,^*^P < 0.05). The tumorigenicity of the fourth-passage spheres and parental SK-UT-1 cells in vivo (**f–g**). The BLAB/C-nu female mice injected with10^5^, 10^6^ parental cells SK-UT-I could not form tumors. Data were presented as mean ± SD (n = 5,^*^P < 0.05)

To further assess the tumor-initiation ability of fourth-passage spheres, the sphere-formation assay was undertaken [20]. As displayed in Fig. 2a, the primary- and fourth-passage spheres could enhance formation of spheres when they were cultured in CSC-M in ultra-low cell-attachment plates, while the fourth-passage spheres remarkably increased sphere-forming rate, indicating that self-renew ability of such spheres was stronger than primary ones.

Furthermore, we assessed the anchorage-independent growth ability of sphere-forming-like cells. As depicted in Fig. 2b, the primary- and fourth-passage spheres could form colonies in soft agar, while the fourth-passage spheres significantly increased colony formation rate, demonstrating that in vitro carcinogenic ability of fourth-passage spheres was enhanced compared with that of primary-passage spheres.

We further compared the migration and invasion abilities of the primary- and fourth-passage spheres using Transwell assay. Figure 2c shows higher migration and invasion rates of the fourth-passage spheres compared with primary-passage spheres.

To determine whether there is a difference in the chemosensitivity of the primary- and fourth-passage spheres, cytotoxicity assay was conducted to assess the cytotoxic effects of DXR. As a result, fourth-passage spheres demonstrated a greater resistant to DXR than the primary-passage spheres under the same conditions (Fig. 2d).

A previous research showed that the increased drug resistance of stem-like cells is partly due to overexpression of the ATP-binding cassette half-transporters (ABCG2) and Oct 4 [21]. We further attempted to measure the expression levels of ABCG2 and Oct4 in the primary- and fourth-passage spheres. It was found that the fourth-passage spheres showed higher expression levels of ABCG2 and Oct4 in comparison with the primary-passage spheres (Fig. 2e). The above-mentioned findings demonstrated that the fourth-passage spheres significantly expressed the characteristics of CSCs in vitro.

To further indicate whether the fourth-spheres have a stronger cancerogenic capability compared with parental cells in vivo, we subcutaneously implanted the fourth-passage spheres derived from SK-UT-1 cells and parental cells with varying number of cells in the tow flanks of nude mice. As shown in Fig. 2f, tumors could be formed with only 10^4^ cells in the fourth-passage spheres, while a minimum of 10^7^ parental cells was required to form xenograft tumors. Moreover, the tumor growth in mice that received fourth-passage spheres was as fast as mice that received 10^7^ parental cells. The time required for reaching the volume of xenografts to approximately 3500–4000 mm^3^ was shorter in mice bearing the fourth-passage spheres than that of mice bearing SK-UT-1 cells (Fig. 2g). Taken together, it was revealed that the fourth-generation spheres have a higher tumorigenic potential, and they may represent a small proportion of cancer stem-like cells in SK-UT-1 cells.

### CD133 ^+^ population demonstrated a great formation of spheres and colonies as well as migration ability

**Fig. 3:**
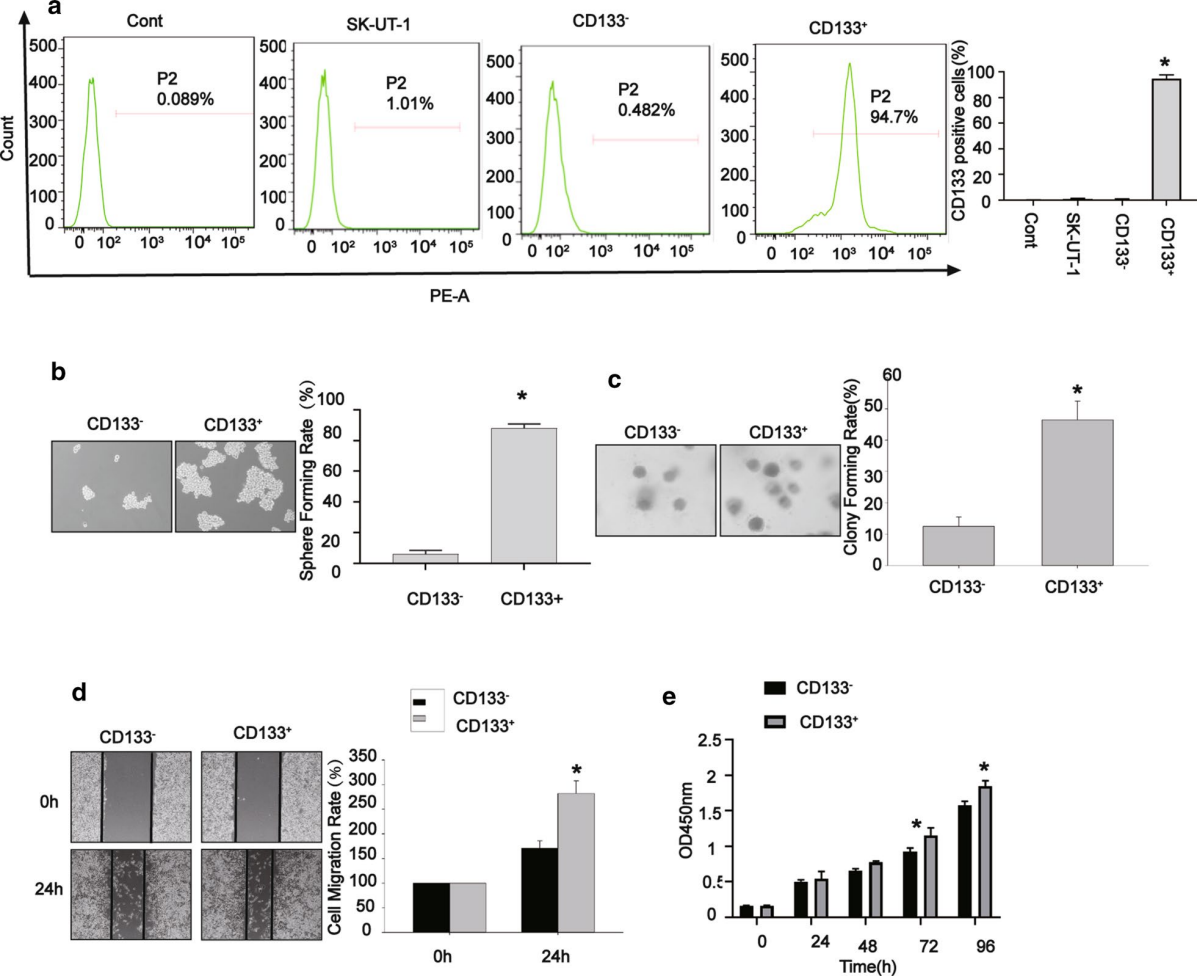
CD133 ^+^ spheres enhanced the carcinogenicity. Sorting of CD133^−^ and CD133^+^ cells by immunomagnetic beads (**a**), CD133^+^ spheres had a higher sphere forming rate (**b**), CD133^+^ spheres had a higher colony forming rate (**c**), CD133^+^ spheres had a higher migration rate (**d**), CD133^+^ spheres exhibited a higher proliferation rate (**e**). Data were presented as mean ± SD (n = 3, *P < 0.05)

Given that cancer stem-like cells are enriched in spheres, the markers predominantly expressed in spheres could be used to identify potential cancer stem-like cells. Our results revealed that spheres derived from SK-UT-1 cells contained a higher expression level of CD133, suggesting the possibility of detecting SK-UT-1-derived cancer stem-like cells using CD133 marker. Thus, we sorted the CD133^−^ and CD133^+^ subpopulations derived from SK-UT-1 cells by immunomagnetic beads. As displayed in Fig. 3a, the percentage of CD133^+^ cells was 94% ± 2.54% for CD133^+^ subpopulation, 1.014% ± 0.004 % for SK-UT-1 cells, and 0.668% ± 0.005% for CD133^−^ subpopulation.

Moreover, we compared the self-renewal and in vitro oncogenic capabilities of CD133^+^ and CD133^−^ subpopulations by the sphere-forming and colony formation assays. As depicted in Fig. 3b, c, the self-renewal and in vitro oncogenic capabilities significantly enhanced in CD133^+^ subpopulation compared with those in CD133^−^ subpopulation, as indicated by increasing sphere-forming rate and colony formation rate. We further investigated the migratory role of CD133^+^ and CD133^−^ subpopulations by wound healing assay. The results showed that the migration rate was higher in CD133^+^ subpopulation than that CD133^−^ subpopulation (Fig. 3d). The cell viability of CD133^+^ subpopulation was more significantly elevated than that of CD133^−^ subpopulation (Fig. 3e), as indicated by CCK-8 assay. Collectively, these data suggested that CD133^+^ subpopulation exhibited a robust formation of spheres and colonies, as well as migration ability, demonstrating that these cells possess stronger properties of cancer stem-like cells compared with CD133^−^ subpopulation derived from SK-UT-1 cells.

### CD133 ^+^ subpopulation demonstrated a great anti-apoptosis ability and high expression levels of cancer stem-like cell- markers

**Fig. 4:**
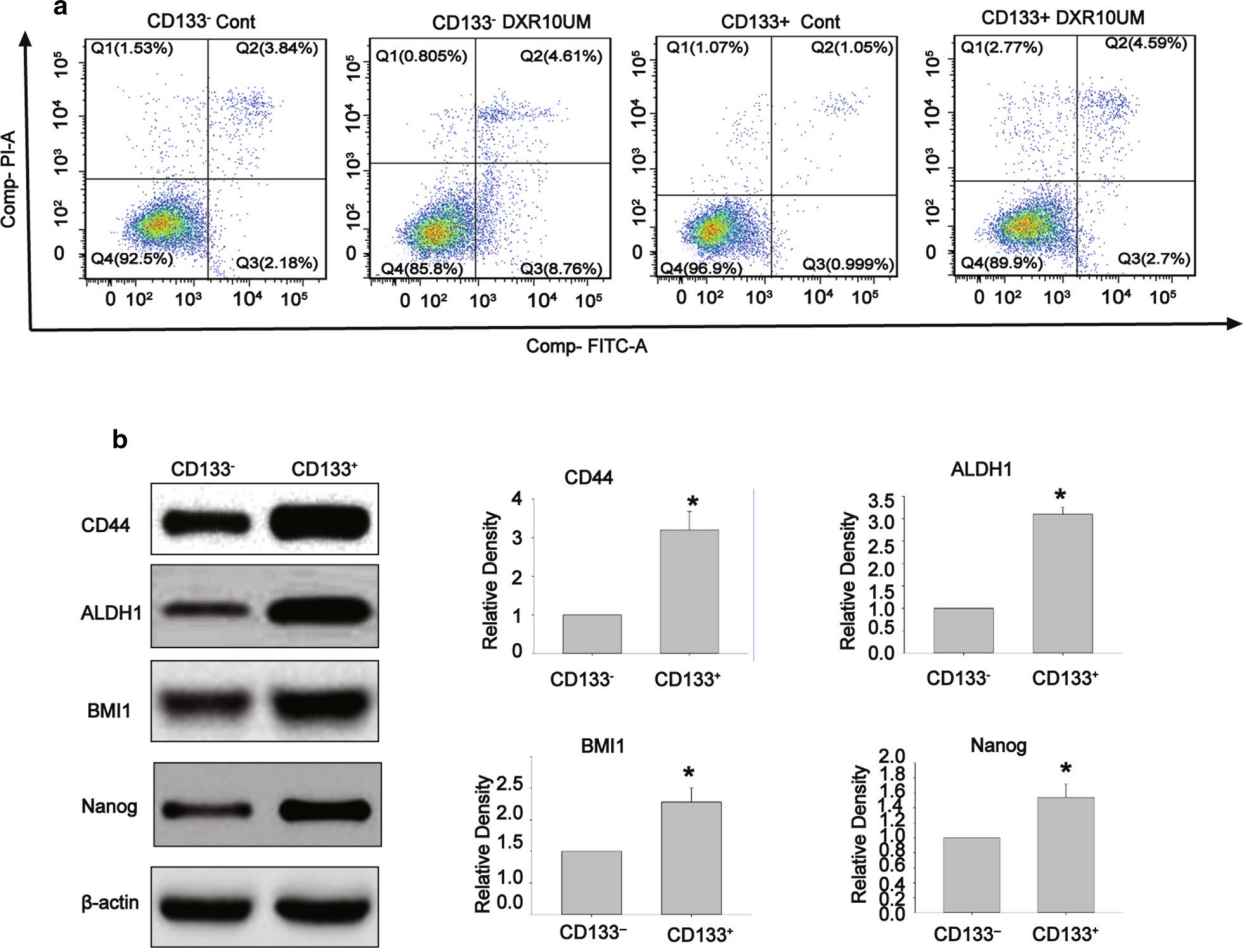
CD133 + spheres had a more robust ability of anti-apoptosis and exhibited higher expression levels of cancer stem-like cells- related markers. The apoptotic rate in CD133^+^ and CD133^−^ subpopulations after treatment with DXR (10 µM) for 24 h (**a**), The total rate of apoptosis (%) = Early apoptosis rate (Q2) + Late apoptosis rate (Q3). the expression levels of CD44, ALDH1, BMI1, and Nanog in CD133^+^ subpopulation and CD133^−^ subpopulation derived from SK-UT-1 cells (**b**). Data were presented as mean ± SD (n = 3,^*^P < 0.05)

In order to indicate whether CD133^+^ subpopulation derived from SK-UT-1 cells has a capability of resistance to apoptosis after treatment with DXR, we compared the apoptotic rate in CD133^+^ subpopulation and CD133^−^ subpopulation treated with DXR (10 µM) for 24 h. As shown in Fig. 4a, CD133^+^ subpopulation demonstrated a stronger capability of spontaneous anti-apoptosis, while resistance to apoptosis after treatment with DXR.

To further characterize the expression levels of CSC-related markers in CD133^+^ subpopulation derived from SK-UT-1 cells, Western blotting was employed to detect the expression levels of CD44, ALDH1, BMI1, and Nanog. Expectedly, we found that CD133^+^ subpopulation had higher expression levels of CD44, ALDH1, BMI1, and Nanog compared with those of CD133^−^ subpopulation (Fig. 4b). Collectively, the above-mentioned results suggested that CD133^+^ subpopulation derived from SK-UT-1 cells possessed capabilities of resistance to apoptosis after treatment with DXR, as well as stemness feature of cancer stem-like cells.

## Discussion

Various types of cancer cells have been found to contain a subset of CSCs or cancer stem-like cells, while little is known about how can we obtain the cancer stem-like cells using an enrichment approach and which molecule can be served as cancer stem cell-surface markers in ULMS. We, in the present research, demonstrated that the fourth-passage spheres derived from SK-UT-1 cells using sphere culture with CSC-M possessed stemness features and enriched the CD133^+^ subpopulation. Correspondingly, the purified CD133 + population displayed stronger capabilities to form spheres and colonies, with resistance to cytotoxic and pro-apoptotic effects after treatment with DXR.

Numerous attempts have been made to identify cells with stemness properties in cancer cell lines. Recently, as a functional approach, sphere formation is particularly significant to enrich potential CSC subpopulations when specific CSC-surface markers have not been defined [22, 23]. To date, no marker for putative ULMS CSCs has been proposed, and therefore, further study is required to explore the isolation method for ULMS CSCs. In the current study, we performed sphere formation to obtain different generations of spheres derived from SK-UT-1 cells. In addition, we found that the primary- and fourth-passage spheres can form spheres when they are cultured in ultra-low cell-attachment plates with CSC-M. Importantly, the fourth-passage spheres increased sphere formation and colony formation rates, migration and invasion abilities, higher expression levels of ABCG2 and Oct4, as well as being more resistant to DXR, indicating that these spheres have stronger stemness features in vitro. Additionally, the percentage of CD133^+^ cells was significantly higher in the fourth-passage spheres compared with that in the primary-passage spheres. More importantly, we demonstrated that the fourth-generation spheres have more tumorigenic potential, and they may represent a small proportion of cancer stem-like cells.

It has been reported that CD133 is dominantly expressed in various types of cancer, and its high expression level is correlated with disease progression, metastasis, recurrence, and poor overall survival in several human malignancies [24, 25]. CD133 plays a potential therapeutic role in cancer stem-like cells. For instance, Waldron et al. [26] conjugated an additional anti-EpCAM scFv converting it to a deimmunized bispecific targeted toxin (dEpCAMCD133KDEL). This bispecific targeted toxin potently inhibited protein translation and proliferation in breast and colon carcinoma cell lines. Finally, dEpCAMCD-133KDEL also caused tumor regression in an in vivo model of head and neck squamous cell carcinoma. Utilizing a bispecific CSC targeted toxin also showed an anti-CSC function in head and neck squamous cell carcinoma in vivo [27]. Zhu et al. [28] employed an AC133-specific chimeric antigen receptor (CAR) and showed that AC133CAR T cells killed AC133 + GBM stem cells (GBM-SCs) both in vitro and in an orthotopic tumor model in vivo. More importantly, when transfecting CD133 mRNA into dendritic cells and vaccinating animals with experimental gliomas, a major histocompatibility complex (MHC)-independent and long-lasting immune response against CD133 was generated [29], and it was revealed that targeting a cell-associated antigen may be an effective strategy to target cancer stem-like cells. However, whether CD133 acts as a maker for cancer stem-like cells remains to be investigated. In the present research, we first sorted the CD133^−^ and CD133^+^ subpopulations derived from SK-UT-1 cells. Meanwhile, the CD133^+^ population in SK-UT-1 cell lines possessed a stronger ability of formation of spheres and colonies, as well as greater migration and proliferation abilities. We further assessed whether the CD133^−^ and CD133^+^ subpopulations derived from SK-UT-1 cells are resistant to spontaneous apoptosis after treatment with DXR. As expected, the CD133^+^ population in SK-UT-1 cells exhibited an enhanced resistance to spontaneous or apoptosis after treatment with DXR. Besides, we detected the expression levels of CSC-related markers in CD133^+^ subpopulation derived from SK-UT-1 cells. It was shown that the expression levels of CD44, ALDH1, BMI1, and Nanog were elevated in the CD133^+^ subpopulation compared with those in CD133^−^ subpopulation. Nonetheless, we observed that the CD133^−^ population reserved a weaker capability for forming spheres and colonies. Thus, we will perform further molecular researches on CD133 to identify cancer stem-like cell population in SK-UT-1 cells.

## Conclusions

In summary, a subset of spheres could serve as cancer stem-like cell population in SK-UT-1 lines. Moreover, CD133 may be a potential marker for SK-UT-1 cells. Our findings may facilitate understanding of mechanisms underlying initiation, development, and progression of ULMS, and present a significant target for the development of therapeutic strategies aiming to improve the prognosis of ULMS via targeting cancer stem-like cells.

## Data Availability

The datasets used and/or analyzed during the current study are available from the corresponding author on reasonable request.

## Abbreviations

CSCs: Cancer stem cells.
CSLCs: Cancer stem cell-like cells.
ULMS: Uterine leiomyosarcoma.
